# Belief Entropy Tree and Random Forest: Learning from Data with Continuous Attributes and Evidential Labels

**DOI:** 10.3390/e24050605

**Published:** 2022-04-26

**Authors:** Kangkai Gao, Yong Wang, Liyao Ma

**Affiliations:** 1 Department of Automation, University of Science and Technology of China, Hefei 230027, China; gkk2010@mail.ustc.edu.cn; 2 School of Electrical Engineering, University of Jinan, Jinan 250022, China; cse_maly@ujn.edu.cn

**Keywords:** decision trees, uncertain data, belief entropy, belief function, random forest, evidential likelihood

## Abstract

As well-known machine learning methods, decision trees are widely applied in classification and recognition areas. In this paper, with the uncertainty of labels handled by belief functions, a new decision tree method based on belief entropy is proposed and then extended to random forest. With the Gaussian mixture model, this tree method is able to deal with continuous attribute values directly, without pretreatment of discretization. Specifically, the tree method adopts belief entropy, a kind of uncertainty measurement based on the basic belief assignment, as a new attribute selection tool. To improve the classification performance, we constructed a random forest based on the basic trees and discuss different prediction combination strategies. Some numerical experiments on UCI machine learning data set were conducted, which indicate the good classification accuracy of the proposed method in different situations, especially on data with huge uncertainty.

## 1. Introduction

Decision trees have been widely used for their good learning capabilities and ease of understanding. In some real world issues, instances may be ill-known for some factors such as randomness, data incompleteness and even expert’s indefinite subjective opinions; however, traditional decision trees can only handle certain samples with precise data. The incompletely observed instances are usually ignored or replaced by a precise one, despite the fact that they may contain useful information [1], which may cause a loss of accuracy.

There have been many attempts to build trees from incomplete data in the past several decades. The probability trees [2,3] were suggested based on probability theory, which is usually intuitively the first tool to modeling uncertainty in practice; however, it has been proven that probability cannot always be adequate for representing data uncertainty [4,5] (often termed epistemic uncertainty). To overcome this drawback, various approaches have been proposed, including: fuzzy decision trees [6,7], the possibilistic decision trees [8] and the uncertain decision trees [9,10]. Besides the aforementioned methods, a more general framework, called the belief function theory [11,12] (also evidential theory or Dempster–Shafer theory), has been proven to have the ability to model all kinds of knowledge. The process of embedding belief functions within decision tree techniques has already been extensively investigated [13,14,15,16,17,18,19,20,21,22,23,24,25] in recent years. Particularly, among these methods, several trees [17,18,19] estimate parameters by maximizing evidential likelihood function using the $E^{2}M$ algorithm [26,27], which is also the basis of part of the trees to be proposed in this paper.

However, the existing methods on incomplete data do not take continuous attributes into full consideration. These proposals deal with uncertain data modeled by the belief function and build trees by extending the traditional decision tree method. The imitation and transformation decides to use existing methods to handle continuous attribute values by discretization, which brings about an issue of losing the detail of the training data. For example, the information gain ratio, the attribute selecting measurement in C4.5, was transformed to adapt the evidential labels of the training set in the Belief C4.5 trees [19], in which the continuous-valued attribute is divided into four intervals of equal width before learning. This issue leads to the purpose of this paper: to learn from uncertain data with continuous attribute values without pretreatment.

To realize this purpose, we firstly, for each attribute, fit the training data to a Gaussian mixture model (GMM), which consists of normal distribution models one-by-one corresponding to class labels, by adopting the $E^{2}M$ algorithm. This step, which significantly differs from other decision trees, confirms the ability to deal with ill-known labels and original attribute values (either discrete or continuous). On the basis of these GMM models, we generate the basic belief assignment (BBA) and calculate belief entropy [28]. The attribute with minimal average entropy, which distinguishes classes from each others most, will be selected as the splitting attribute. The following decision tree induction steps are designed accordingly and logically. To our knowledge, this paper is the first to introduce GMM models and belief entropy to decision trees with evidential data.

Another part of our proposal is adopting the ensemble method for our belief entropy trees. Inspired by the idea of building bagging trees based on random sampling [29], we further choose a more efficient and popular technique—random forest [30]. Under the belief function framework, the basic trees will output either precise or mass (modeled by BBA) label predictions, while traditional random forest can only combine precise labels. Thus, a new method to summarize the basic tree predictions is proposed to combine mass labels directly, instead of voting on precise labels. This combined mass keeps the uncertain information of data as much as possible, which helps to generate a more reasonable prediction. The new combination method is discussed and compared to the traditional majority voting method later.

We note that we have proposed our early work in a shorter conference paper [31]. Compared with our initial conference paper, we have fixed the attribute selection and splitting strategy of a single tree and introduced ensemble learning to our tree method in this paper.

Section 2 recalls some basic knowledge about decision trees, belief function theory, the $E^{2}M$ algorithm and belief entropy. Section 3 details the induction procedure of belief entropy methods and proposes three different instance prediction techniques. In Section 4, we introduce how to expend the single belief entropy tree to random forests and discuss the different predicting combination strategies. In Section 5, we detail experiments that were carried out on some classical UCI machine learning data sets to compare the classification accuracies of proposed trees and random forests. Finally, conclusions are summarized in Section 6.

## 2. Settings and Basic Definitions

The purpose of a classification method is to build a model that maps an attribute vector $X=\left(x^{1},\ldots ,x^{D}\right)\in A^{1}\times A^{2}\times \cdots \times A^{D}$, which contains D attributes, to an output class $y\in C=\left\{C_{1},\ldots ,C_{K}\right\}$ taking its value among K classes. Each attribute discretely has finite values or continuously takes value within an interval. The learning of classification is based on a complete training set of precise data which contains *N* instances, denoted as

$$T=\left(\begin{array}{c} X_{1},y_{1}\\ \vdots \\ X_{N},y_{N} \end{array}\right)=\left(\begin{array}{c} x_{1}^{1},\ldots ,x_{1}^{D},y_{1}\\ \vdots \\ x_{N}^{1},\ldots ,x_{N}^{D},y_{N} \end{array}\right).$$

However, the imperfect knowledge about the inputs (feature vector) and the outputs (classification labels) exists widely in practical applications. Traditionally and regularly, the imperfect knowledge is modeled by probability theory, which is considered to be questionable in a variety of scenarios. Hence, we model uncertainty by belief function in this paper. Typically, we consider that attribute values are precise and can be either continuous or discrete, while only the output labels are uncertain.

### 2.1. Decision Trees

Decision trees [32] are regarded as one of the most effective and efficient machine learning methods and widely adopted for solving classification and regression problems in practice. The success, to a great extent, relays on the easily understandable structure, for both humans and computers. Generally, a decision tree is induced top-down from a training set *T*, which recursively repeats the steps below:

- Select an attribute, through a designed selection method, to generate a partition of a training set;
- Split the current training set to several subsets and put them into child node;
- Generate a leaf node and determine the prediction label for a child node when a stop criterion is satisfied.

Differing in the attribute selection methods, several decision tree algorithms have been proposed, such as ID3 [32], C4.5 [33] and CART [34]. Among these trees, the ID3 and C4.5 choose entropy as an information measure to compute and evaluate the quality of a node split by a given attribute.

The core of ID3 is information gain. Given training data *T* and an attribute *A* with $K_{A}$ modalities, the information gain will be:

(1) $$Gain\left(T,A\right)=Info\left(T\right)-Info_{A}\left(T\right)$$

where

(2) $$Info\left(T\right)=-\sum_{i=1}^{K}\theta_{i}\log_{2}\left(\theta_{i}\right)$$

and

(3) $$Info_{A}\left(T\right)=-\sum_{i=1}^{K_{A}}\frac{\left|T_{i}\right|}{\left|T\right|}Info\left(T_{i}\right)$$

where $\theta_{i}$ is the proportion of instances in *T* that are of class $C_{i}$, $\left|T\right|$ and $\left|T_{i}\right|$ are the cardinalities of the instance sets belonging to a parent node and to the child node *i*.

The limitation of information gain is that attributes with largest values will be most promoted  [33], which leads to the GainRatio in the C4.5 algorithm. It is given as:

(4) $$GainRatio\left(T,A\right)=\frac{Gain\left(T,A\right)}{SplitInfo\left(T,A\right)}$$

where

(5) $$SplitInfo\left(T,A\right)=-\sum_{i=1}^{K_{A}}\frac{\left|T_{i}\right|}{\left|T\right|}\log_{2}\frac{\left|T_{i}\right|}{\left|T\right|}.$$

The attribute with the largest gain ratio will be selected for splitting.

We can easily find the the Equation (2) is actually the Shannon Entropy. Yet in this paper, concerning the feature of evidential data described by the framework of belief function, the attribute selection method is newly designed based on belief entropy [28] instead of Shannon entropy.

### 2.2. Random Forest

To improve the classification accuracy and generalization ability of machine learning, the ensemble model method is introduced to the learning procedure. One important branch of ensemble method is called bagging, which concurrently builds multiple basic models learning from different training sets, which are generated from original data by bootstrap sampling. On the basis of bagging decision trees, random forest (RF) [30] not only chooses the training instance randomly but also introduces randomness into attributes selection. To be specific, traditional decision trees select the best splitting attribute among all *D* attributes; random forest generates a random attribute subset then chooses the best one within this subset to split the tree node. The size $D^{\prime }$ of this subset is adjustable and generally set as $D^{\prime }=log_{2}D$.

A detailed description of the mathematical formulation of RF model is found in [30]. The RF model consists of a union of multiple basic trees, where each tree learns from bootstrap samples and selects attribute from a small subset of all attributes. There some advantages of RF: (a) better prediction performance, (b) resistance to overfitting, (c) low correlation of individual trees, (d) low bias and low variance and (e) small computational overhead.

Some existing works have explored the ensemble method on belief decision trees, such as bagging  [29]. In this paper, we apply the random forest technique to the proposed belief entropy trees and discuss the different prediction determining strategies.

### 2.3. Belief Function Theory

Let the finite set Ω denote the frame of discernment containing *k* possible exclusive values that a variable can take. When considering the output *y*, the imperfect knowledge about value of *y* can be modeled by mass function $m_{y}:2^{\Omega }\to \left[0,1\right]$, such that $m_{y}\left(⌀\right)=0$, and

(6) $$\sum_{A\subseteq \Omega }m_{y}\left(A\right)=1,$$

which is also called a basic belief assignment (BBA). The subset *A* is called a focal set where $m_{y}\left(A\right)>0$, and the $m_{y}\left(A\right)$ can be interpreted as the support degree of the evidence towards the case that true value is in set *A*.

There are some typical mass functions need to be attended:

- Vacuous mass: mass function such that $m_{y}\left(\Omega \right)=1$, which means total ignorance;
- Bayesian mass: for all focal set *A*, the cardinality $\left|A\right|=1$. In this case, the mass degenerates to a probability distribution;
- Logical(categorical) mass: $m_{y}\left(A\right)=1$ for some *A*. In this case, the mass is equivalent to the set *A*.

One-to-one related to the mass function $m_{y}$, the belieffunction and plausibilityfunction are defined as:

(7) $$Bel_{y}\left(B\right)=\sum_{A\subseteq B}m_{y}\left(A\right),$$

(8) $$Pl_{y}\left(B\right)=\sum_{A\cap B\neq ⊘}m_{y}\left(A\right),$$

which, respectively, indicate the minimum and maximum belief degree of evidence towards set *B*. Typically, the function $pl:\Omega \to \left[0,1\right]$ such that $pl_{y}\left(\omega \right)=Pl_{y}\left(\left\{\omega \right\}\right)$ for all ω∈Ω is called contourfunction associated to $m_{y}$.

For two mass function $m_{1}$ and $m_{2}$ induced by evidences independently, they can be combined by the $Dempster^{\prime }s\;rule$ [12]⊕ defined as:

(9) $$\left(m_{1}⊕m_{2}\right)\left(A\right)=\frac{1}{1-\kappa }\sum_{B\cap C=A}m_{1}\left(B\right)m_{2}\left(C\right),$$

for all A⊆Ω,A≠⌀, and $\left(m_{1}⊕m_{2}\right)\left(⌀\right)=0$, where

(10) $$\kappa =\sum_{B\cap C=⌀}m_{1}\left(B\right)m_{2}\left(C\right),$$

is called the degreeofconflict between $m_{1}$ and $m_{2}$. Obviously, Dempster’s rule is commutative and associative according to the definition.

In the decision making situation, we need to determine the most reasonable hypothesis from a mass. Different decision-making strategies with belief functions [35,36] have been researched. Among these methods, in the transferable belief model (TBM), pignisticprobability [37] was proposed to make decision from a BBA:   

(11) $$BetP\left(\omega \right)=\sum_{A\subseteq \Omega ,\omega \in A}\frac{m\left(A\right)}{\left|A\right|},$$

where $\left|A\right|$ is the cardinality of *A*.

When we model uncertain labels of evidential data with mass functions, the training set becomes

$$T=\left(\begin{array}{c} X_{1},m_{1}\\ \vdots \\ X_{N},m_{N} \end{array}\right)=\left(\begin{array}{c} x_{1}^{1},\ldots ,x_{1}^{D},m_{1}\\ \vdots \\ x_{N}^{1},\ldots ,x_{N}^{D},m_{N} \end{array}\right).$$

### 2.4. Evidential Likelihood

Consider a discrete random vector *Y* taking values in Ω with a probability mass function $p_{Y}\left(y;\theta \right)$ assumed to be associated with a parameter θ∈Θ. After a realization *y* of *Y* has been perfectly observed, the likelihood function of completedata is defined as $L:\Theta \to \left[0,1\right]$ such that

(12) $$L\left(\theta ;y\right)=p_{Y}\left(y;\theta \right),\forall \theta \in \Theta .$$

When the observations are uncertain, it is impossible to evaluate parameter θ from a likelihood function. In this situation, a new statistical tool [38] called evidential likelihood was proposed to perform parameter estimation. Assume that *y* is not precisely observed, but is known surely that y∈A for some A∈Ω. Given such imprecisedata, the likelihood function will be extended to

(13) $$L\left(\theta ;A\right)=p_{Y}\left(A;\theta \right)=\sum_{y\in A}p_{Y}\left(y;\theta \right),\forall \theta \in \Theta .$$

Furthermore, the observation of instance *y* could be not only imprecise, but also uncertain, which is modeled by mass function $m_{y}$. Thus the evidential likelihood function [27] can be defined as

(14) $$\begin{array}{cc} L\left(\theta ;m_{y}\right) & =\sum_{A\subseteq \Omega }L\left(\theta ;A\right)m_{y}\left(A\right)\\ \; & =\sum_{y\in \Omega }p_{Y}\left(y;\theta \right)\sum_{A∋x}m_{y}\left(A\right)\\ \; & =\sum_{y\in \Omega }p_{Y}\left(y;\theta \right)pl\left(y\right),\forall \theta \in \Theta \end{array},$$

where the pl is the contour function related to $m_{y}$ and the $L\left(\theta ;m_{y}\right)$ can be remarked as $L\left(\theta ;pl\right)$. According to the statement of Denoeux [27], the value $1-L\left(\theta ;pl\right)$ equals to the conflict between parametric model $p_{Y}\left(y;\theta \right)$ and the uncertain observation pl, which means minimizing $L\left(\theta ;pl\right)$ is actually a procedure of estimating the best parameter θ to fit the parametric model to observation as closely as much.

Equation (14) also indicates that $L\left(\theta ;pl\right)$ can be remarked as the expectation of pl such that

(15) $$L\left(\theta ;pl\right)=E_{\theta }\left[pl\left(Y\right)\right].$$

Assume that $Y=\left(y_{1},\ldots ,y_{N}\right)$ is a sample set containing n cognitively independent [12] and i.i.d. uncertain observations, in which the $y_{i}$ is model by $m_{y_{i}}$. In the situation the Equation (15) is written as a product of *n* terms:

(16) $$L\left(\theta ;pl\right)=\prod_{n=1}^{N}E_{\theta }\left[pl_{n}\left(y_{n}\right)\right].$$

### 2.5. E${}^{2}$M Algorithm

Though an extension of likelihood function, the maximum likelihood estimation of evidential likelihood can not directly be computed by the broadly applied EM algorithm [39]. The $E^{2}M$ algorithms [27] introduced by Denoeux allow us to maximize the evidential likelihood iteratively, which is composed of two steps (similar to EM algorithm):

1. The **E-step** require firstly a probability mass function $p_{Y}\left(\cdot |pl;\theta^{\left(q\right)}\right)=p_{Y}\left(\cdot ;\theta^{\left(q\right)}\right)⊕pl$, in which the former part means the probability mass function of *Y* under the parameter $\theta^{\left(q\right)}$ estimated from last iteration and the latter part indicates contour function pl. The expression is:
  (17) $$p_{Y}\left(y|pl;\theta^{\left(q\right)}\right)=\frac{p_{Y}\left(y;\theta^{\left(q\right)}\right)pl\left(y\right)}{L\left(\theta^{\left(q\right)};pl\right)}.$$
  Then calculate the expectation of log likelihood $\log L_{c}\left(\theta ;y\right)=\log p_{Y}\left(y;\theta \right)$ of complete data with respect to $p_{Y}\left(\cdot |pl;\theta^{\left(q\right)}\right)$,
  (18) $$Q\left(\theta ,\theta^{\left(q\right)}\right)=\frac{\sum_{y\in \Omega }\log\left(L_{c}\left(\theta ;y\right)\right)p_{Y}\left(y;\theta^{\left(q\right)}\right)pl\left(y\right)}{L\left(\theta^{\left(q\right)};pl\right)}.$$
2. The **M-step** is to maximize $Q\left(\theta ,\theta^{\left(q\right)}\right)$ with respect to θ, obtaining a new estimation that ensures $Q\left(\theta^{\left(q+1\right)},\theta^{\left(q\right)}\right)⩾Q\left(\theta ,\theta^{\left(q\right)}\right)$.

The two steps repeat until $L\left(\theta^{\left(q+1\right)}\right)-L\left(\theta^{\left(q\right)}\right)⩽\epsilon$, where ϵ is a set threshold.

### 2.6. Belief Entropy

Inspired by Shannon entropy [40], which can measure uncertainty contained by a probability distribution, a type of belief entropy called Deng entropy is proposed by Deng [28] to handle situation where the traditional probability theory is limited. When the uncertain information is described by the basic belief assignment instead of the probability distribution, Shannon entropy cannot work. Deng entropy is defined on the belief function frame, which makes it able to measure uncertain information described by the BBA efficiently.

Let *A* be the focal set of belief function, and $\left|A\right|$ be the cardinality of *A*. Deng entropy *E* is defined as:

(19) $$E\left(m\right)=-\sum_{A\subseteq \Omega }m\left(A\right)\log\frac{m\left(A\right)}{2^{\left|A\right|}-1}.$$

We can easily learn from the definition that if the mass function is Bayesian, which means $\left|A\right|=1$ for all *A*, Deng entropy degenerates to Shannon entropy such that

(20) $$E\left(m\right)=-\sum_{A\subseteq \Omega }m\left(A\right)\log m\left(A\right).$$

The greater the cardinality of the focal set is, the bigger the corresponding Deng entropy is, so that the evidence imprecisely refers to more single elements. Thus, significant Deng entropy indicates huge uncertainty. Powered by this feature, we calculate the average Deng entropy of BBAs to select the best attribute leading to the least uncertainty. The details are shown in the next section.

## 3. Design of Belief Entropy Trees

Up to now, various decision tree methods have been proposed to deal with evidential data, but many of them consider categorical attributes and transform the continuous attribute values into discrete categories. Some recent works fit the continuous attributes with same class labels into normal distributions [41] and generate BBA from the normal distributions to select the best splitting attribute by calculating belief entropy [42]; however, this method divides samples into each set of certain classes, which can only handle the precise class labels. Our goal is to develop a belief decision tree method learns from data set with continuous and precise attribute values but incomplete class labels directly and efficiently.

This section explains our method in detail, specifically focusing on the procedure of attribute selection. Corresponding splitting strategy, stopping criterion and the leaf structure are also well-designed to accomplish the whole belief entropy decision tree.

### 3.1. The Novel Method to Select Attribute

The learning procedure of decision trees is generally to decide the split attribute and to decide how to split on this attribute on each node; our method also proceeds in this manner. As a novel decision tree, the most characteristic and core part of our method is the attribution selection, which includes three steps: firstly, for each attribute, fit the values to normal distributions corresponding to each class label, in another words, fit attribute values into K×D normal distribution models, where K is the class number and D is the attributes number of instances; secondly, for every instance, generate D BBAs from each attribute according to the normal distribution-based models; finally, calculate belief entropy from BBAs for each attribute. The attribute with minimum belief entropy will be selected to split.

#### 3.1.1. Parameter Estimation on Data with Continuous Attributes

Powered by the idea of extracting BBAs from normal distribution-modeled attribute values [41], we try to operate similarly on data with ill-known class labels. In the situation that each instance exactly belongs to one class, the d-th attribute values set $\left\{x_{1}^{d},\ldots ,x_{N}^{d}\right\}$ is divided into K subsets $\left\{x_{n}^{d}|y_{n}=C_{k}\right\},k=1,\ldots ,K$ corresponding to each class. It is easy to fit each subset to the normal distribution by calculating means and standard deviations.

**Figure 1:**
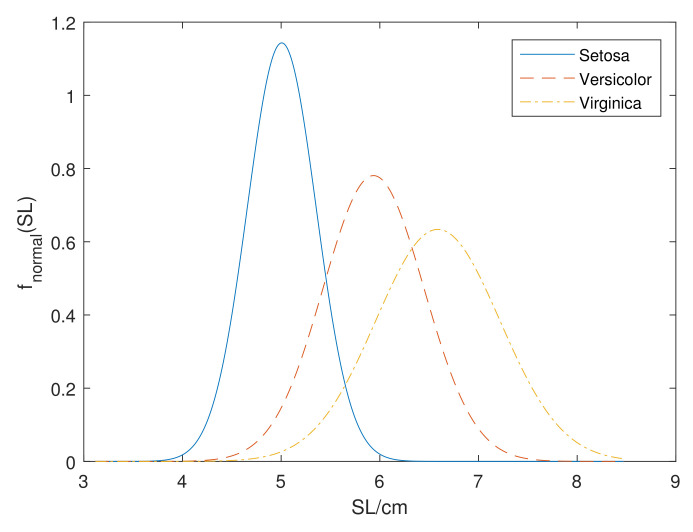
The normal distribution df three classes for the SL attribute of Iris data set.

**Example** **1.** *Consider the Iris data set [43], a classical machine learning data set, which contains 150 training instances of three classes: ‘Setosa’, ‘Versicolor’, ‘Virginica’, with four attributes: sepal length(SL), sepal width(SW), petal length(PL) and petal width(PW). For the values of attribute SL in the class of Setosa, we can directly calculate the mean value as μ=5.0133 and standard deviation as σ=0.3267. Similarly, we can obtain normal distribution parameters of class of Versicolor and Virginica. Figure 1 shows the normal distribution model of Iris data set for the SL attribute in three classes.*

However, when the labels of training set are ill-known, some instances can not be allocated to a certain class assertively. The evidential likelihood and $E^{2}M$ algorithm introduced in Section 2 make it possible to generate an estimation of model parameters. Because the $E^{2}M$ algorithm uses only contour functions, the label of n-th instance will be represented by plausibility $pl_{n}=\left\{pl_{nk}\right\},k=1,\ldots ,K$ instead of mass function $m_{n}$.

For the purpose of comparing attributes, we split the whole training data into D attribute–label pairs and handle D parameter estimation problems. Consider the *d*-th attribute value vector $X^{d}={\left(x_{1}^{d},\cdots ,x_{N}^{d}\right)}^{T},d\in \left\{1,\ldots ,D\right\}$, we assume the conditional distribution of $X^{d}$ when given $y=C_{k}$ is normal with mean $\mu_{k}$ and standard deviation $\sigma_{k}$:

$$X^{d}|\left(y=C_{k}\right)∼N\left(\mu_{k},\sigma_{k}^{2}\right),k=1,\ldots ,K.$$

Actually the assumption is to build a one-dimensional Gaussianmixturemodel(GMM) [44]. Similar to the application of $E^{2}M$ algorithm in lineardiscriminantanalysis [45], the following discuss is practically to adopt $E^{2}M$ algorithm to estimate parameters in GMM.

Let $\pi_{k}$ be the marginal probability when $y=C_{k}$, and $\theta =(\mu_{1},\ldots ,\mu_{K},\sigma_{1},\ldots ,\sigma_{K},\pi_{1},\ldots ,\pi_{K})$ the parameter vector to be estimated. The complete-data likelihood is

(21) $$\begin{array}{cc} L_{c}\left(\theta \right) & =\prod_{n=1}^{N}p\left(x_{n}^{d}\left|Y_{n}=y_{n}\right.\right)p\left(y_{n}\right)\\ & =\prod_{n=1}^{N}\prod_{k=1}^{K}\phi {\left(x_{n}^{d};\mu_{k},\sigma_{k}\right)}^{y_{nk}}\pi_{k}^{y_{nk}}, \end{array}$$

where the ϕ is normal distribution probability density,

(22) $$\phi \left(x;\mu ,\sigma \right)=\frac{1}{\sqrt{2\pi }\sigma }e^{-\frac{{\left(x-\mu \right)}^{2}}{2\sigma^{2}}},$$

and $y_{nk}$ is a binary indicator variable, such that $y_{nk}=1$ if $y_{n}=C_{k}$ and $y_{ik}=0$ if $y_{N}\neq C_{k}$.

when expended to evidential data, where we use contour function to describe the labels, the evidential likelihood is drew from Equation (16) that,

(23) $$\begin{array}{cc} L\left(\theta \right) & =\prod_{n=1}^{N}E_{\theta }\left[pl_{n}\left(y_{n}\right)\right]\\ \; & =\prod_{n=1}^{N}\sum_{k=1}^{K}pl_{nk}\phi \left(x_{n}^{d};\mu_{k},\sigma_{k}\right)\pi_{k}. \end{array}$$

According to the $E^{2}M$ algorithm, we compute the expectation of complete-data log likelihood

(24) $$\ell_{c}\left(\theta \right)=\log L_{c}\left(\theta \right)=\sum_{n=1}^{N}\sum_{k=1}^{K}y_{nk}\left[\log\phi \left(x_{i}^{d};\mu_{k},\sigma_{k}\right)+\log\pi_{k}\right]$$

with respect to the combined mass probability function

(25) $$p\left(x^{d}\left|pl;\theta^{\left(q\right)}\right.\right)=\prod_{n=1}^{N}p\left(x_{n}^{d}\left|pl_{n};\theta^{\left(q\right)}\right.\right).$$

To simplify the equation, we denote

(26) $$\zeta_{nk}^{\left(q\right)}=p\left(x_{n}^{d}\left|pl_{n};\theta^{\left(q\right)}\right.\right)=\frac{pl_{nk}\pi_{k}^{\left(q\right)}\phi \left(x_{n}^{d};\mu_{k}^{\left(q\right)},\sigma_{k}^{\left(q\right)}\right)}{\sum_{k=1}^{K}pl_{nk}\pi_{k}^{\left(q\right)}\phi \left(x_{n}^{d};\mu_{k}^{\left(q\right)},\sigma_{k}^{\left(q\right)}\right)}.$$

Finally, we obtain the to-be-maximized function

(27) $$Q\left(\theta ,\theta^{\left(q\right)}\right)=\sum_{n=1}^{N}\sum_{k=1}^{K}\zeta_{nk}^{\left(q\right)}\left[\log\phi \left(x_{n}^{d};\mu_{k},\sigma_{k}\right)+\log\pi_{k}\right]$$

in the E-step.

The formal of $Q\left(\theta ,\theta^{\left(q\right)}\right)$ is similar to the function computed in the EM algorithm on the GMM [44]. Because of the similarity, we imitate it and learn that the optimal parameter maximizing $Q\left(\theta ,\theta^{\left(q\right)}\right)$ can be iteratively computed by

(28) $$\pi_{k}^{\left(q+1\right)}=\frac{1}{n}\sum_{n=1}^{N}\zeta_{nk}^{\left(q\right)},\;\mu_{k}^{\left(q+1\right)}=\frac{\sum_{n=1}^{N}\zeta_{nk}^{\left(q\right)}x_{i}^{d}}{\sum_{n=1}^{N}\zeta_{nk}^{\left(q\right)}},$$

(29) $$\sigma_{k}^{\left(q+1\right)}=\sqrt{\frac{\sum_{n=1}^{N}\zeta_{nk}^{\left(q\right)}{\left(x_{n}^{d}-\mu_{k}^{\left(q+1\right)}\right)}^{2}}{\sum_{n=1}^{N}\zeta_{nk}^{\left(q\right)}}}$$

Finally when $L\left(\theta^{\left(q+1\right)}\right)-L\left(\theta^{\left(q\right)}\right)⩽\epsilon$ is satisfied for some ϵ, stop the iteration and remark $\theta^{\left(q+1\right)}$ as $\theta^{d}=\left(\mu_{1}^{d},\ldots ,\mu_{K}^{d},\sigma_{1}^{d},\ldots ,\sigma_{K}^{d},\pi_{1}^{d},\ldots ,\pi_{K}^{d}\right)$, which is the estimation of parameters in the GMM extracted from d-th attribute. Repeat this procedure for every attributes of the training set, D×K normal distribution

$$N_{k}^{d}\left(\mu_{k}^{d},{\sigma_{k}^{d}}^{2}\right),d=1,\ldots ,D,k=1,\ldots ,K$$

will be generated.

The Algorithm 1 shows the procedure of parameter estimation and there is Example 2 to help understand it.
**Algorithm 1** Parameter estimation of GMMs.**Input:** evidential training set $T_{pl}=\left(x,pl_{y}\right)$, iteration stop threshold ϵ**Output:** estimated normal distribution parameter matrix $\left(\mu_{k}^{d},\sigma_{k}^{d}\right),d=1,\ldots ,D,k=1,\ldots ,K$1:**for** each attribute $A^{d}$ **do**2: initialize parameters as ${\theta^{d}}^{\left(0\right)}=\left({\mu_{1}^{d}}^{\left(0\right)},\ldots ,{\mu_{K}^{d}}^{\left(0\right)},{\sigma_{1}^{d}}^{\left(0\right)},\ldots ,{\sigma_{K}^{d}}^{\left(0\right)},{\pi_{1}^{d}}^{\left(0\right)},\ldots ,{\pi_{K}^{d}}^{\left(0\right)}\right)$;3: q=0; Initialize loop variable.4: **for** *q* **do**5:   update the estimation of parameters ${\theta^{d}}^{\left(q+1\right)}=\left({\mu_{1}^{d}}^{\left(q+1\right)},\ldots ,{\mu_{K}^{d}}^{\left(q+1\right)},{\sigma_{1}^{d}}^{\left(q+1\right)},\ldots ,{\sigma_{K}^{d}}^{\left(q+1\right)},{\pi_{1}^{d}}^{\left(q+1\right)},\ldots ,{\pi_{K}^{d}}^{\left(q+1\right)}\right)$ by Equations (28) and (29).6:   **if** $L\left({\theta^{d}}^{\left(q+1\right)}\right)-L\left({\theta^{d}}^{\left(q\right)}\right)⩽\epsilon$ **then**7:    break; {End the loop if evidential likelihood increment is less than threshold.}8:   **end if**9:   q=q+1;10:  **end for**11:  adopt $\left({\mu_{k}^{d}}^{\left(q+1\right)},{\sigma_{k}^{d}}^{\left(q+1\right)}\right),k=1,\ldots ,K$ as estimated normal distribution parameters under attribute $A^{d}$;12:**end for**

**Figure 2:**
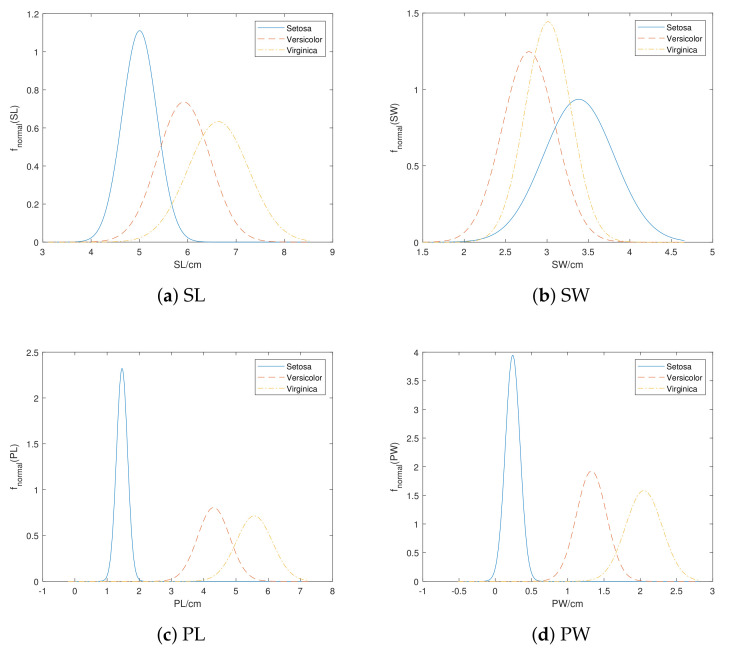
The normal distribution models for each attribute in evidential Iris data set.

**Example** **2.** *Consider the Iris data set mentioned in Example 1. To simulate the situation that labels of training set are not completely observed, we manually introduce uncertainty to the Iris data. In this example, we set that each instance has an equivalent chance (25%) to be vacuous, imprecise, uncertain or completely observed (the detail of transformation is discussed in Section 5). Table 1 shows the attribute values and labels described by plausibility pl of some instances in evidential Iris data. Table 2 shows the mean and standard deviation pairs $\left(\mu ,\sigma \right)$ calculated by $E^{2}M$ algorithm. Figure 2 shows curves of these models.*

#### 3.1.2. BBA Determination

This step is to generate D BBAs corresponding to each attribute for every instance in the training set.

Choose an instance $I_{n}$ with attribute vector $x_{n}=\left(x_{n}^{1},\ldots ,x_{n}^{D}\right)$ from the data set, calculate the intersection of $x_{n}^{d}\left(d=1,\ldots ,D\right)$ and the K normal distribution functions $\phi_{k}^{d}=\phi \left(x^{d};\mu_{k},\sigma_{k}\right),k=1,\ldots ,K$, i.e., we obtain K normally distributed probability density function (PDF) values for the attribute $A^{d}$ and instance $I_{n}$, denoted as $\phi_{nk}^{d},k=1,\ldots ,K$.

Due to the property that the probability of a value x sampling from a normal distribution is proportional to the PDF $\phi \left(x\right)$, we can infer, for the attribute d, the probability that instance $x_{n}$ belongs to each class is proportional to $\phi_{nk}^{d}=\phi \left(x_{n}^{d};\mu_{k},\sigma_{k}\right),k=1,\ldots ,K$. From this opinion of statistical analysis, the rule to assign normal PDFs to some sets was proposed to build BBAs.

Firstly, normalize the $\phi_{nk}^{d}$ with different class *k* such that

(30) $$f_{k}=\phi_{nk}^{d}/\sum_{k=1}^{K}\phi_{nk}^{d}.$$

Then rank $f_{k}$ in decreasing order $f_{r}^{\prime }\left(r=1,\ldots ,K\right)$, whose corresponding class is denoted as $C_{r}^{\prime }\left(r=1,\ldots ,K\right)$. Assign $f_{r}^{\prime }$ to the class set by the following rule:

(31) $$\begin{array}{cc} \; & m\left(\left\{C_{1}^{\prime }\right\}\right)=f_{1}^{\prime }\\ & m\left(\left\{C_{1}^{\prime },C_{2}^{\prime }\right\}\right)=f_{2}^{\prime }\\ & \cdots \\ & m\left(\left\{C_{1}^{\prime },\ldots ,C_{K}^{\prime }\right\}\right)=m\left(\theta \right)=f_{K}^{\prime }. \end{array}$$

If $f_{i}^{\prime }=f_{i+1}^{\prime }=\ldots =f_{j}^{\prime }$, then $m\left(\left\{C_{1}^{\prime },\ldots ,C_{j}^{\prime }\right\}\right)=\sum_{p=i}^{j}f_{p}^{\prime }$. By this rule, we obtain a nested BBA of $x_{n}$ under the select attribute $A^{d}$, which we denote as $m_{n}^{d}$.

**Example** **3.** *Consider the first instance of the evidential Iris data set showed in Table 1, whose attributes are:*

$$x^{SL}=5.1,\;x^{SW}=3.5,\;x^{PL}=1.4,\;X^{PW}=0.2.$$

**Figure 3:**
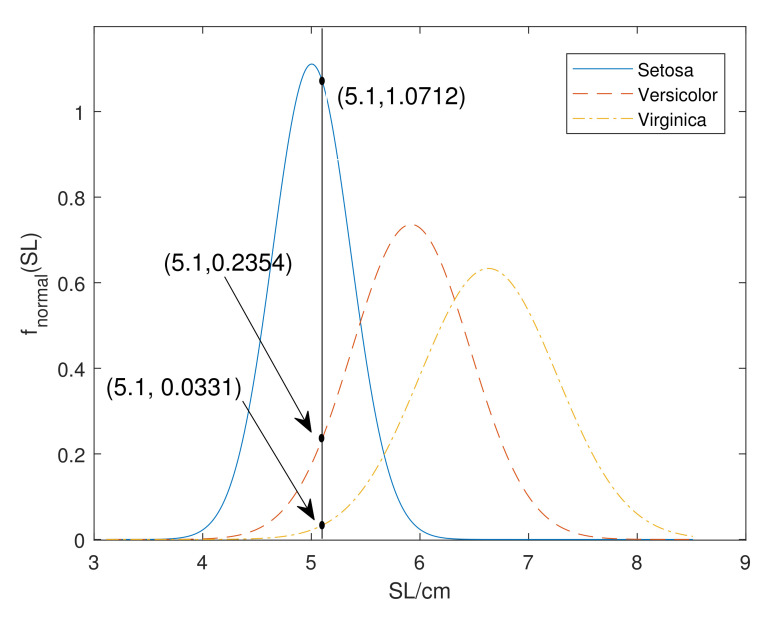
The normal distribution for the SL attribute in three classes.

*For attribute SL, the intersections of $x^{SL}=5.1$ and three normal distributions are shown in Figure 3 such that*

$$\begin{array}{cc} \; & \phi_{Setosa}^{SL}\left(x^{SL}\right)=1.0712,\\ \; & \phi_{Versicolor}^{SL}\left(x^{SL}\right)=0.2354,\\ \; & \phi_{Virginica}^{SL}\left(x^{SL}\right)=0.0331. \end{array}$$

*The reader can see in the figure that this instance is closest to class ‘Setosa’, then to the ‘Versicolor’ and ‘Virginica’. Thus, we generate BBA from intersection values, which is intuitive. The BBA is assigned as:*

$$\begin{array}{cc} \; & m\left(\left\{Setosa\right\}\right)=\frac{1.0712}{1.0712+0.2354+0.0331}=0.7996\\ \; & m\left(\left\{Setosa,Versicolor\right\}\right)=\frac{0.2354}{1.0712+0.2354+0.0331}=0.1757\\ \; & m\left(\left\{Setosa,Versicolor,Virginica\right\}\right)=\frac{0.0331}{1.0712+0.2354+0.0331}=0.0247 \end{array}$$

*Similarly, we build BBAs for the rest of the attributes—shown in Table 3.*

#### 3.1.3. Calculation of Belief Entropy

The last step to determine splitting attribute is to calculate the average Deng entropy

(32) $$E^{d}=E\left(A^{d}\right)=\frac{1}{N}\sum_{n=1}^{N}E\left(m_{n}^{d}\right),d=1,\ldots D$$

of all instances for each attribute. As mentioned in Section 2.6, Deng entropy measures the uncertain degree contained by BBA, which means the less $E^{d}$, the more certainty the BBAs contain, and the more separate the division of classes is. Consequently, we choose the attribute $A^{*}$ that minimizes the average Deng entropy such that

(33) $$A^{*}=\underset{A^{d}}{\arg\min}\left(E\left(A^{d}\right)\right),d=1,\ldots ,D$$

to be the best splitting attribute to proceed the tree building.

**Example** **4.** *Continue the Examples 2 and 3. Calculate Deng entropy of BBAs of selected instance shown in Table 3:*

$$\begin{array}{cc} E^{SL}\left(m\right) & =-0.7996\times \log\frac{0.7996}{2^{1}-1}-0.1757\times \log\frac{0.1775}{2^{2}-1}-0.0247\times \frac{0.0247}{2^{3}-1}=0.2933\\ E^{SW}\left(m\right) & =-0.6904\times \log\frac{0.6904}{2^{1}-1}-0.2329\times \log\frac{0.2329}{2^{2}-1}-0.0767\times \frac{0.0767}{2^{3}-1}=0.3687\\ E^{PL}\left(m\right) & =-1\times \log\frac{1}{2^{1}-1}=0\\ E^{PW}\left(m\right) & =-1\times \log\frac{1}{2^{1}-1}=0 \end{array}$$

*Similarly proceed same calculation to all instances so that average Deng entropy for attributes are calculated that*

$$E\left(A^{SL}\right)=1.3853,\;E\left(A^{SW}\right)=2.0837,\;E\left(A^{PL}\right)=0.4275,\;E\left(A^{PW}\right)=0.2116.$$

*According to this result, attribute PW will be chosen to generate child nodes.*

*Comparing the Deng entropy with the curves in Figure 2, we can intuitively learn that PW has the most distinctive curves for each class, yet curves in SW overlap each other a lot, which conforms to the size of the average Deng entropy above, where PW is the lowest and SW is the highest.*

As a matter of fact, Examples 1–4 in this chapter can be orderly combined as a whole calculating example, which shows the procedure of the proposed attribute selecting method.

### 3.2. Splitting Strategy

The splitting strategy is redesigned according to the selected attribute $A^{*}$ to fit the proposed attribute selection method. Branches will be associated to each class, that is to say, each node to be edged will have K branches. For an instance $I_{n}$, consider the generated BBAs, the class corresponding to the maximum mass value will be the branch that this instance shall be put into. To put it simply, when splitting the tree under attribute $A^{*}$, the instance $I_{n}$ will be assigned into the $k_{n}$-th child node, where the $k_{n}$ satisfies

(34) $$k_{n}=\underset{k}{\arg\max}\phi_{nk}^{*}\left(x_{n}^{d}\right).$$

The Algorithm 2 summarizes the procedure of selecting attribute and splitting. It should be mentioned that, though the child nodes are associated to each class, this splitting strategy does not mean to determine the affiliation of instances directly and arbitrarily in this step.
**Algorithm 2** Attribute selection and splitting.**Input:** evidential training set $T_{pl}=\left(x,pl_{y}\right)$, possible splitting attribute $A=\left\{A^{1},\ldots ,A^{D}\right\}$**Output:** selected attribute $A^{*}$, instance sets in child nodes $T_{i}$,$\left(i=1,\ldots ,K\right)$1:compute the normal distribution parameters $\left(\mu_{k}^{d},\sigma_{k}^{d}\right)$ for each $A^{d}$ and $C_{k}$ by $E^{2}M$ algorithm;2:**for** each attribute $A^{d}$ **do**3: **for** each instance $I^{n}$ **do**4:  generate BBA $m_{n}^{d}$ from normal distributions $N_{k}^{d}\left(\mu_{k}^{d},{\sigma_{k}^{d}}^{2}\right),k=1,\ldots ,K$;5:  $E_{n}^{d}=E\left(m_{n}^{d}\right)$; {Calculate Deng entropy for all generated BBAs}6: **end for**7:  $E\left(A^{d}\right)=Average\left(E_{n}^{d}\right)$;  {Calculate average Deng entropy for each attribute}8:**end for**9:split on attribute $A^{*}=\underset{A^{d}}{\arg\min}\left(E\left(A^{d}\right)\right)$; {The attribute with minimum average entropy is selected}

### 3.3. Stopping Criterion and Prediction Decision

After designing the attribute selection and partitioning strategy, we split each decision node to several child nodes. This procedure repeats iteratively until one of the stop criterion is met:

- No more attributes for selection;
- The number of instances in the nodes falls below a set threshold;
- The labels of instances are all precise and fall into the same class;

When the tree building stops at a leaf node *L*, a class label should be determined to predict the instances that fall into this node. We design two different prediction methods such that:

- The first one is to generate the prediction label from the original training labels of instances contained by this node, which is a similar treatment to traditional decision trees such as C4.5 tree method. Denoting the instances in the leaf node by $\left\{I_{1}^{\prime },\ldots I_{P}^{\prime }\right\}$ and the corresponding evidential training labels by $\left\{pl_{p}^{\prime }\right\},p=1,\ldots P$, the leaf node will be labeled by $\hat{C}$, where
  (35) $$\hat{C}=\underset{C_{k}}{\arg\max}\sum_{p=1}^{P}pl_{p}^{\prime }\left(C_{k}\right),k=1,\ldots ,K,$$
  which means the class label with maximal plausibility summation will represent this node. This tree predicts from the original labels of training set, which is called Oringin-predictionbeliefentropytree(OBEtree) for short in this paper.
- The first method described above in fact abandons the generated BBAs during the tree build procedure, which will be adopted to generating predicted instance label in the second method. Firstly, the splitting attributes list, which lead instance $I^{\prime }$ to the leaf node from top to down, are denoted by $\left\{A_{1}^{*},\ldots ,A_{Q}^{*}\right\}$, and the BBAs generated accordingly are denoted by $m_{1}^{*}\ldots ,m_{Q}^{*}$. Then combine these BBAs by Dempster rule, such that $\hat{m}=m_{1}^{*}⊕\cdot \cdot \cdot ⊕m_{Q}^{*}$, to predict the training instance. On this basis, we continue to combine generated BBAs of all instances in a leaf node such that ${\hat{m}}_{leaf}={\hat{m}}_{1}⊕\cdot \cdot \cdot ⊕{\hat{m}}_{P}$, where the once again combined BBA ${\hat{m}}_{leaf}$ will be the mass prediction label for the whole leaf node. To obtain a precise label for another choice, the last step is making decision on BBA by choosing the class label with maximal pignistic probability computed by Equation (11). We call this tree a Leaf-predictionbeliefentropytree(LBEtree) in this paper.

The Algorithm 3 summarizes the induction of belief entropy trees introduced in this section.
**Algorithm 3** Induction of belief entropy trees (BE-tree).**Input:** evidential training set $T_{pl}$, classifier type TYPE**Output:** belief entropy tree Tree1:construct a root node containing all instances $T_{p}l$;2:**if** stopping criterion is met **then**3: **if** TYPE=OBE **then**4:  output precise prediction generated from original plausibility label for the whole node;5: **else if** TYPE=LBE **then**
6:  combine BBAs generates during each splitting $\hat{m}=m_{1}^{*}⊕\cdot \cdot \cdot ⊕m_{Q}^{*}$ for each instance;7:  combine BBAs of all instances in previous node generated in step 6 that ${\hat{m}}_{leaf}={\hat{m}}_{1}⊕\cdot \cdot \cdot ⊕{\hat{m}}_{P}$;8:  output ${\hat{m}}_{leaf}$ as a mass prediction for the whole leaf node;9:  output $\hat{C}=Pignistic\left({\hat{m}}_{leaf}\right)$ as a precise prediction for the whole leaf node;10: **end if**11: return Tree=root node;12:**else**13: apply Algorithm 2 to select splitting attribute $A^{*}$;14: induce each subset ${T_{pl}}_{child}$ based on $A^{*}$;15: **for** all ${T_{pl}}_{child}$ **do**16:   $Tree_{child}=BE$-$tree\left({T_{pl}}_{child}\right)$; {Recursively build the tree on the new child node}17:   attach $Tree_{child}$ to the corresponding Tree;18:  **end for**19:**end if**

### 3.4. An Alternative Method for Predicting New Instance

Two types of belief entropy trees, the OBE tree and the LBE tree, have been described in detail in the last section. Similar to traditional decision trees, a new instance will be classified in a top-down way: starting at the root node and following branches by considering its generated BBA under splitting attribute until reaching a leaf node. The prediction of leaf node will be given to this new instance.

However, differing from the idea of collecting the numerous ‘opinions’ of instances, another method to predict a new instance is considered after a tree has been built. In Section 3.1.2, we introduced how to generate each training instance’s BBA corresponding to attributes. In the same way, we can generate $m_{1}^{*}\ldots ,m_{Q}^{*}$ corresponding to an attributes list $\left\{A_{1}^{*},\ldots ,A_{Q}^{*}\right\}$, which orderly splits and leads the new instance to a leaf node. Then, we combine these BBAs such that $\hat{m}=m_{1}^{*}⊕\cdot \cdot \cdot ⊕m_{Q}^{*}$ to predict the new testing instance. It is easy to find that this method performs the same way as the front part of label prediction in LBE trees, yet stops when obtaining a mass prediction from the testing instance’s own attribution values instead of the leaf node it belongs to, which also means testing instances in a same leaf node normally have different mass prediction under this design. For the sake of narrative, a tree predicting in this way is called Instance-predictionbeliefentropytree(IBEtree) in this paper.

**Figure 4:**
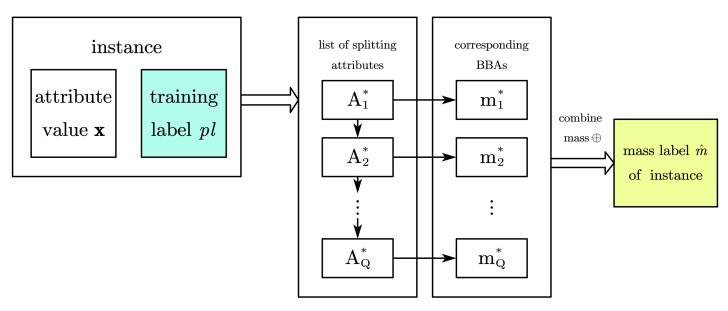
Generation of mass prediction for each instance.

**Figure 5:**
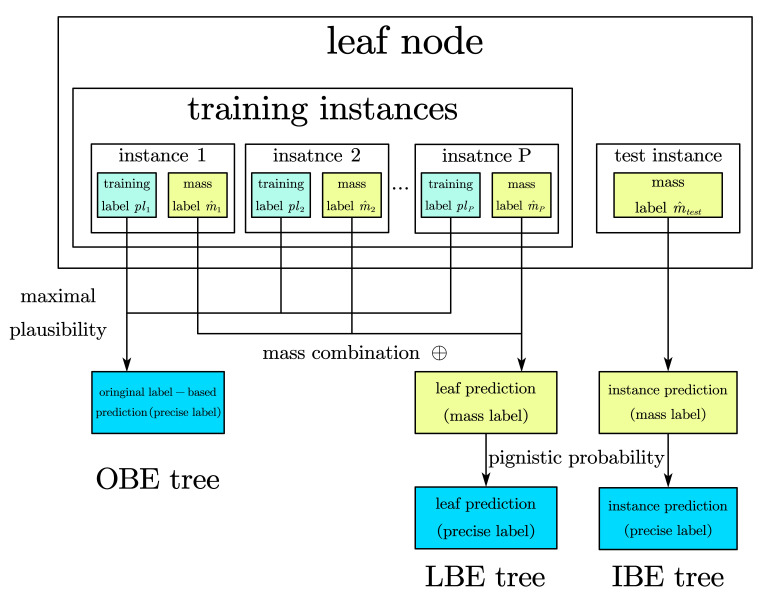
Three different ways to make prediction in belief entropy trees.

Figure 4 and Figure 5 show the procedure of making prediction on leaf node, where Figure 4 is the generation of mass prediction $\hat{m}$ for each instance, in whether training set or testing set; Figure 5 details the different prediction making in the proposed three belief entropy trees.

## 4. Belief Entropy Random Forest

We have introduced the induction of belief entropy trees in the previous section, which is regarded as the basic classifier of random forest ensemble method in the following discussion.

The generalization ability of random forest draws from not only the perturbation of sampling, but also the perturbation of attributes selecting. Specific to the proposed belief entropy random forest, for each basic tree, we firstly performs bootstrap sampling on the original training set, which means randomly sampling with replacement for *N* times on the set *T* where $\left|T\right|=N$. Secondly, when training on this resampling set, for each to-be-split node, the best splitting attribute will be chosen from a subset $\left\{A_{i}^{\prime }\right\},i=1,\ldots ,D^{\prime }$ of the set of all available attributes $\left\{A_{j}\right\},j=1,\ldots ,D$, where $1<D^{\prime }<D$. If $D^{\prime }=D$, the basic tree splits totally, the same as the belief entropy tree; while $D^{\prime }=1$ means randomly selecting an attribute to split all the time.

Repeat the first and second steps above *S* times then a ‘forest’ containing variable basic trees will be constructed, where the repeat time *S* is called forest size. When making a prediction of a new instance on this forest, *S* primary predictions will be independently generated by *S* basic trees and finally summarized to one result. It should be mentioned that the OBE tree output precise label directly for testing instances while the LBE trees and IBE trees can provide mass labels described by BBAs or precise labels. This feature inspires two different strategies for making predictions in the last step: the majority voting for precise labels and belief combination of mass labels.

Algorithm 4 shows the procedure of building the complete evidential random forests based on belief entropy trees. Selecting different ensemble prediction strategies and base tree types, we build five random forest lists below:

- label-voting OBE Random Forest(L-OBERF), which performs majority voting on precise outputs of OBE trees;
- label-voting LBE Random Forest(L-LBERF), which performs majority voting on precise outputs of LBE trees;
- mass-combination LBE Random Forest(M-LBERF), which combines BBAs generated by LBE trees and makes decision;
- label-voting IBE Random Forest(L-IBERF), which performs majority voting on precise outputs of IBE trees;
- mass-combination IBE Random Forest(M-IBERF), which combines BBAs generated by IBE trees and makes decision;

**Algorithm 4** Building procedure of evidential random forests.**Input:** evidential training set $T_{pl}$, new instance x, base classifier number *h*, base classifier type TYPE, base classifier output mode *O***Output:** predicted label $\hat{y}$1: **for** i=1:h **do**2: $T_{i}=RandomAttributeSampling\left(RandomInstanceSampling\left(T_{pl}\right)\right)$; {The resampling procedure of each base tree.}3: **if** TYPE=OBE **then**4:  $Tree_{i}=OBE\left(T_{i}\right)$;
5: **else if** TYPE=IBE **then**
6:  $Tree_{i}=IBE\left(T_{i}\right)$; 7. **else if** TYPE=LBE **then**
8:  $Tree_{i}=LBE\left(T_{i}\right)$;9: **end if**10:**end for**11:**for** i=1:h **do**12: $L_{i}=Label\;Prediction\left(Tree_{i},x\right)$; {Generate predict labels of each base tree.}13:**end for**14:**if** O=preciselabel **then**15: $\hat{y}=Majority\left(L_{1},\ldots ,L_{h}\right)$; {Generate prediction from precise labels.}
16:**else if** O=masslabel **then**
17: $\hat{y}=Pignistic\left(MassCombination\left(L_{1},\ldots ,L_{h}\right)\right)$; {Generate prediction from mass labels.}18:**end if**

**Figure 6:**
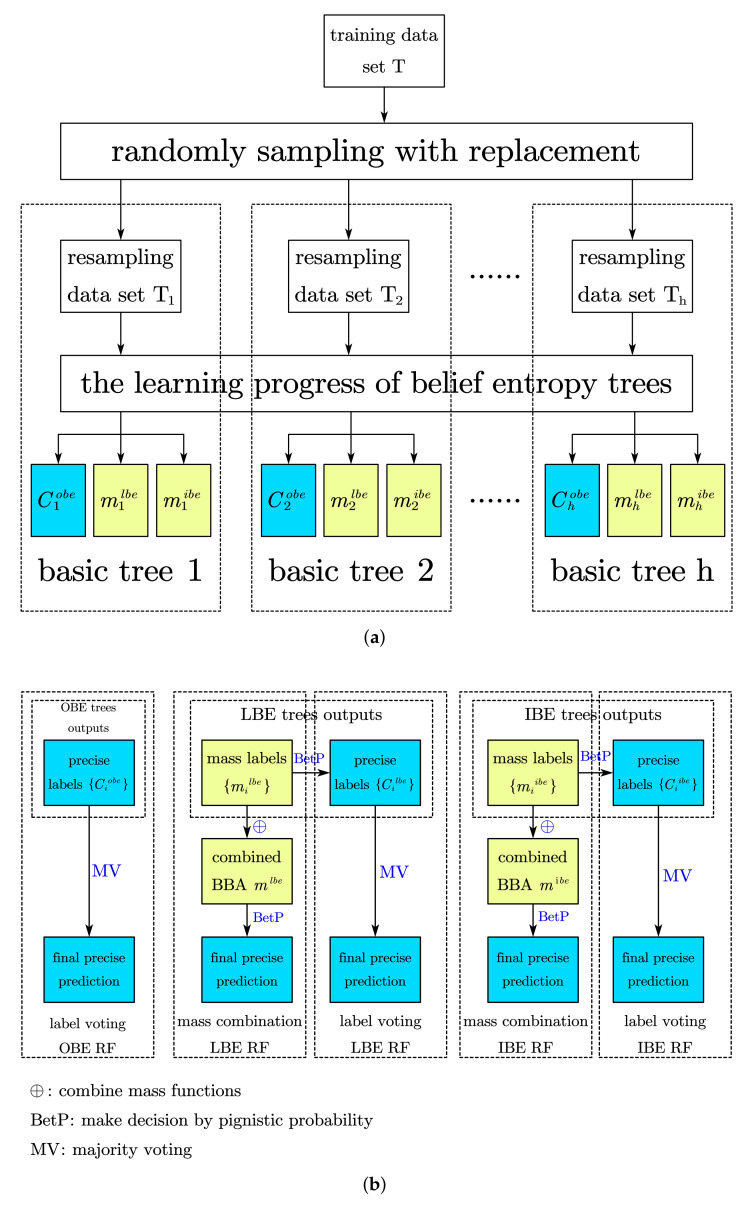
Belief entropy in random forests. (**a**) Generation of basic trees and their outputs in random forest. (**b**) Combination strategies of different random forests.

Figure 6 shows the procedure of constructing the forests, in which the Figure 6a shows generation of basic trees in a random forest, and Figure 6b shows different procedure of combining the final prediction in five forests, which will lead to a different classification performance. We will evaluate them in the next section.

## 5. Experiments

In this section, we detail experiments to evaluate the performance of the proposed decision tree method. The experiment settings and results are detailed below.

### 5.1. Experiment Settings

As there are no widely accepted evidential data sets to measure the proposed method, it is necessary to generate a data set with ill-known labels from machine learning databases taken from the UCI repository [46]. We selected several data sets, including: Iris, Wine, Balance scale, Breast cancer, Sonar and Ionosphere.

Denote the true label of a instance by $C_{i}$, and give its uncertain observation $m_{y_{i}}$. Due to the characters of belief function, we can simulate several situations from precise data:

• a precise observation is such that $pl_{y_{i}}\left(C_{i}\right)=1$, and $pl_{y_{i}}\left(C_{j}\right)=0,\forall C_{j}\neq C_{i}^{*}$;
• a vacuous observation is such that $pl_{y_{i}}\left(C_{j}\right)=1,\forall C_{j}\in C$;
• an imprecise observation is such that $pl_{y_{i}}\left(C_{j}\right)=1$ if $C_{j}=C_{i}^{*}$ or $C_{j}\in C_{rm}$, and $pl_{y_{i}}\left(C_{j}\right)=0$ otherwise, where $C_{rm}$ is a set of randomly selected labels;
• an uncertain observation is such that $pl_{y_{i}}\left(C_{i}^{*}\right)=1$, and $pl_{y_{i}}\left(C_{j}\right)=r_{j},\forall C_{j}\neq C_{i}^{*}$, where $r_{j}$ are sampled independently from uniform distribution $U\left(\left[0,1\right]\right)$.

To observe the performance on evidential training data sets with different ill-known types and incomplete degrees, we set three variables, vacuousness level $V\in \left[0,1\right]$, imprecision level $I\in \left[0,1\right]$ and uncertainty level $U\in \left[0,1\right]$, to adjust the generation procedure, where V+I+U⩽1.

Example 2 shows the transformed Iris data set and listed part of instances in Table 1. In this example, labels of no.53 and no.54 instance are vacuous; labels of no.1 and no.2 instance are imprecise; labels of no.4 and no.52 instance are uncertain.

To improve the reliability and reduce the stochasticity, we performed 5-fold cross-validation on each data set and repeat ten times to compute an average classification accuracy for all experiments. Different tree induction techniques will be compared:

- traditionalC4.5tree, which only uses precise data in the training set during tree induction;
- beliefentropytrees described in Section 3.3: OBE tree, LBE tree, IBE tree;
- beliefentropyrandomforest described in Section 4:
  - label-votingOBErandomforest(L-OBERF);
  - label-votingLBErandomforest(L-LBERF);
  - mass-combinationLBErandomforest(M-LBERF);
  - label-votingIBErandomforest(L-IBERF);
  - mass-combinationIBErandomforest(M-IBERF);

We set the maximal size of the leaf node as $\left|T\right|/20$ to avoid overfitting in the belief entropy trees. In the random forests, the forest size was set as 50, and the size of the attributes subset was set as $D^{\prime }=log_{2}D$.

### 5.2. Experiments on Vacuous Data

**Figure 7:**
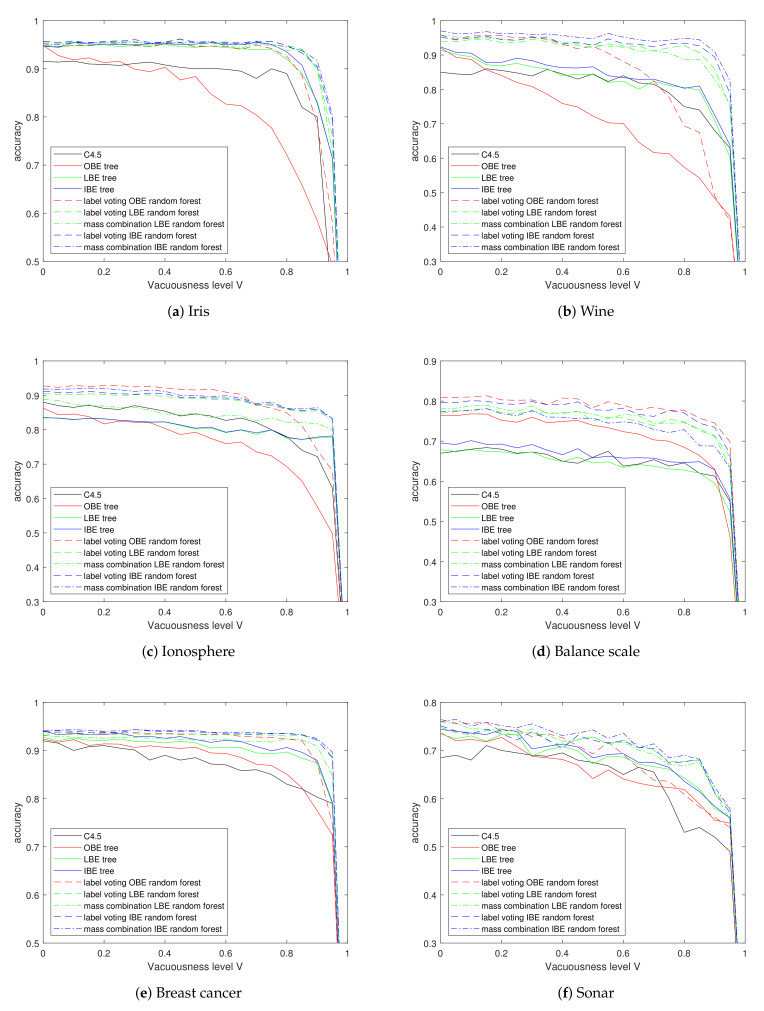
Classification accuracy on UCI data sets with different vacuousness levels.

Assuming part of the instances in the training set are totally unobserved while others are completely observed, we performed experiments with different vacuousness levels $V\in \left[0,1\right]$ while I=U=0. Generating the training sets and learning on them, the results are shown in Figure 7.

Firstly, we observe the figure as a whole. Obviously, whatever the tree induction method is, it is impossible to learn from data sets whose instances are all vacuous. Thus, the accuracy of all trees decreases gradually as *V* increases, yet drops sharply when the *V* approaches nearly to 1. On the contrary, almost all curves keep steady or decrease slightly before the vacuousness level reaches 80%, except for the OBE trees. Table 4 shows the accuracy results when V equals 90%.

Considering the basic belief entropy trees firstly, the LBE trees and IBE trees perform, most of the time, at least as well as the traditional C4.5 decision trees, and better than the traditional decision trees for some time, especially when encountering high vacuousness level *V*; however, the OBE preforms elusively on different data sets: it has the lowest classification accuracy in Iris, Wine and Ionosphere data set; however, it achieves better results in the Balance scale. It is possible that if all samples in a leaf node are vacuous, the direct combination of all the training labels stays vacuous, which led to the shortage of OBE tree.

It can be observed that the belief entropy random forests perform well overall for their improvement in classification accuracy compared to the corresponding basic tree and the slower accuracy decent rate as *V* increases. Among these forests, the ones based on IBE and making prediction by mass combination performs better than others in nearly all data sets except the Balance scale.

### 5.3. Experiments on Imprecise Data

The second situation is that some data are imprecisely observed, i.e., the observation is a set value, while the true value lies in this set (called superset labels [47] in some works). As mentioned before, imprecision level *I* controls the percentage of imprecise observations.

**Figure 8:**
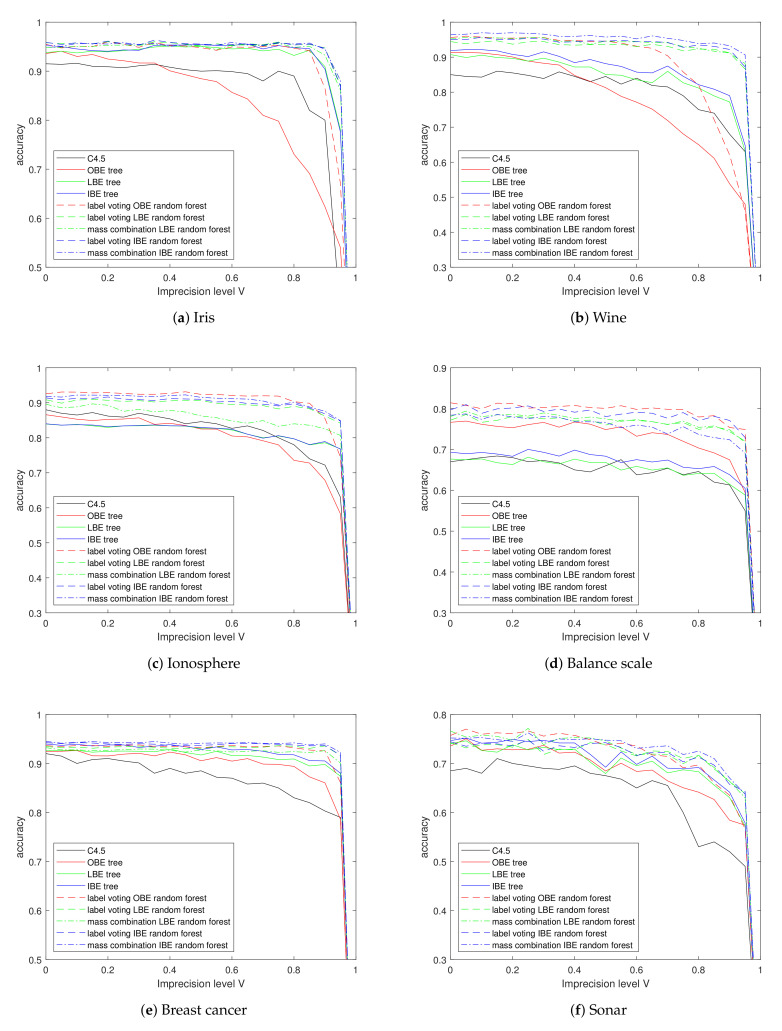
Classification accuracy on UCI data sets with different imprecision levels.

For the instance to be imprecise, we randomly generate a number $z_{k}\in \left[0,1\right]$ for each class $C_{k}$ except the true one. Plausibility of labels with $z_{k}<I$ will be set to 1. When the I=1, a training set becomes totally imprecise, which is, in practice, the same situation as total vacuousness; while I<1, instances are in a middle state of transition from precise to vacuous, which indicates a piece of similarity between the vacuous training set and the imprecise training set, i.e., we can tell that the imprecise sample contains more information than the totally vacuous ones. As a result, we can see in Figure 8, that curves of accuracy with changing I are similar to those in experiments with vacuousness in Figure 7, yet more smooth and full.

According to the Table 5, the proposed methods keep pretty good classification results under high-level imprecise observations. OBE still keeps the shortage in almost all data sets while LBE and IBE achieve similar performance. *M*-IBERF keeps its advantage in most situations, especially in the Iris and Breast Cancer data; the classification accuracy is almost equal to the results on the total precise training set. The balance scale is a particular case to be discussed later.

### 5.4. Experiments on Uncertain Data

Another type of ill-known label is the uncertain one, which is measured by a plausibility distribution, with the true label having the highest chance among all class labels. To evaluate the performance of the proposed trees and forests in a more general situation with uncertainty, we set $U\in \left[0,1\right]$ and V=I=0. For instance, to be transformed into an uncertain one, we assign a value 1 to the plausibility of the true label and random values averagely sampled from $\left[0,U\right]$ to other labels.

**Figure 9:**
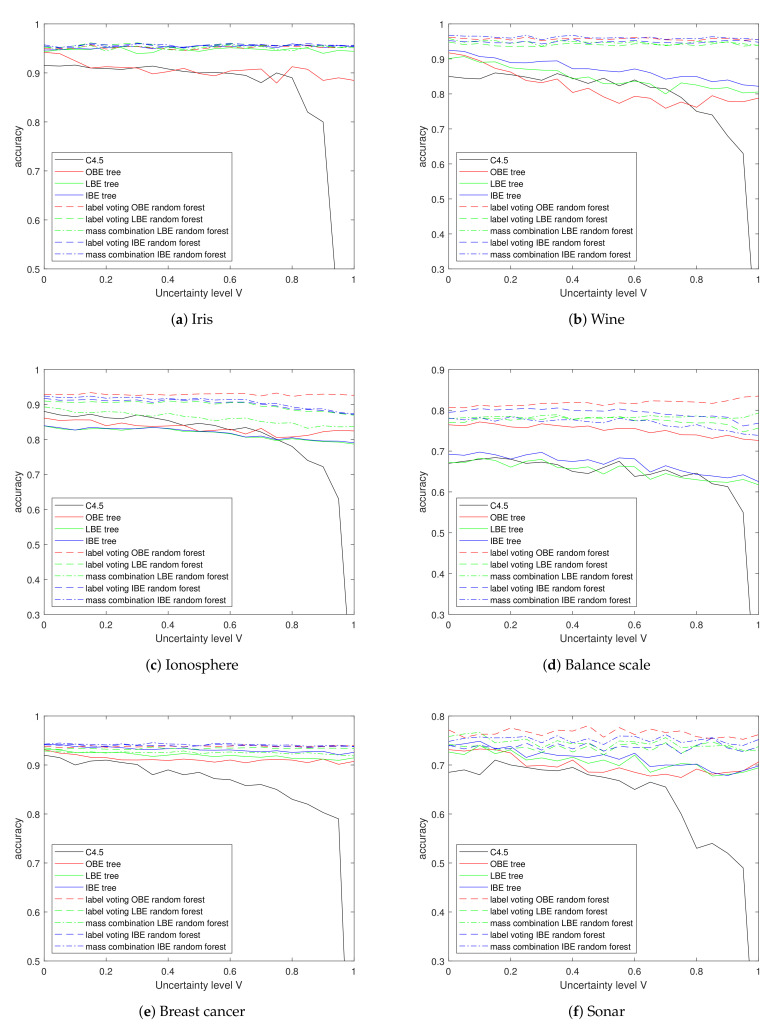
Classification accuracy on UCI data sets with different uncertainty levels.

Despite the inability to handle total vacuousness and imprecision, the belief entropy trees have the ability to learn from totally uncertain training data sets. The horizontal curves in Figure 9 indicate all methods proposed in this paper keep stable performance with changing uncertainty level *U*. On the whole, we can learn from the figure that LBE and IBE perform equally well and better than OBE as a single tree in most data sets, except in the Balance scale.

Considering the forests, for the good attribute normality of Iris, Wine and Breast cancer data, classification accuracies of the five forests on these data sets have similar performance according to Table 6, leading to a heavy overlap of curves in figure. Among these trees, the OBE trees achieve the most significant improvement by building random forest; this improvement helps OBE-RF to surpass other forests in the Ionosphere, Sonar and Balance scale data sets. Particularly, in the Balance scale, the accuracy of OBE-RF even increases slightly as the *U* decreases, which can be partially explained by the fact that uncertain instances are more informative then absolutely precise instances.

### 5.5. Summary

By carrying out experiments on training sets with different types and degrees of incomplete observation, we can conclude that the LBE trees and IBE trees, along with four types of random forests based on them, generally possess excellent learning ability on data with ill-known labels. Among the RFs, the ensemble of the IBE tree, L-IBE-RF and M-IBE RF achieve the highest classification accuracy in most situations except on samples with high uncertainty levels, especially on the Balance scale data set. We think there are two reasons: (a) compared to vacuous and imprecise samples, the learning labels of uncertain samples are more information rich, while the OBE use the learning labels to predict directly; (b) the attribute values of Ionosphere, Balance, and Sonar data sets contain less normality than others—the balance scale are totally not normal. We can conclude that the ensemble OBE RF requests less normality of the data set.

The results of experiments indicate that the application of the belief function tool to the prediction of trees and combination of forests is efficient and reasonable; yet there are also some drawbacks. Firstly, the introduction of the belief function and mass combination obviously increases the time cost of learning. The sensitivity to the normality of data makes the proposed trees and RFs unable to handle, to the greatest extent, all situations with one particular structure.

## 6. Conclusions

In this paper, a new classification tree method based on belief entropy is proposed to cope with uncertain data. This method directly models continuous attribute values of training data by $E^{2}M$ algorithm, and selects a splitting attribute via a new tool–belief entropy. Differing from the traditional decision trees, we redesign the splitting and prediction, making them fit the feature of uncertain labels described by the belief function. Finally, random forests with different combination strategies were constructed on the basis of the proposed tree method to seek higher accuracy and stronger generalization ability.

As the experimental results show, the proposed belief entropy trees are robust to different sorts of uncertainty. They perform closely to traditional decision trees on precise data and keep good results on data with ill-known labels. Meanwhile, the belief entropy random forests, which improve significantly when compared to the basic belief function trees, achieve excellent and stable performance even in the situation with high-level uncertainty. It is proved that the proposed trees and random forests have a potentially broad field of application. In future research, some further improvements will be investigated, such as more reasonable BBA combination methods for the incapacity of Dempster’s rule to handle huge mass conflict, and a boosting ensemble method based on the belief entropy trees.

## Figures and Tables

**Table 1:** Uncertain instances in evidential Iris data set.

Number	Attributes				Contour Functions			True Label
	SL	SW	PL	PW	pl (Setosa)	pl (Versicolor)	pl (Virginica)	
1	5.1	3.5	1.4	0.2	1	0	1	Setosa
2	4.9	3.0	1.4	0.2	1	0	1	Setosa
3	4.7	3.2	1.3	0.2	1	0	0	Setosa
4	4.6	3.1	1.5	0.2	1	0.7498	0.4073	Setosa
…								
51	7.0	3.2	4.7	1.4	0	1	0	Versicolor
52	6.4	3.2	4.5	1.5	0.9519	1	0.7087	Versicolor
53	6.9	3.1	4.9	1.5	1	1	1	Versicolor
54	5.5	2.3	4.0	1.3	1	1	1	Versicolor
…								
101	6.3	3.3	6.0	2.5	0	0	1	Virginica
102	5.8	2.7	5.1	1.9	0.4458	0.5088	1	Virginica
103	7.1	3.0	5.9	2.1	0	0	1	Virginica
104	6.3	2.9	5.6	1.8	1	1	1	Virginica
…								
150	5.9	3.0	5.1	1.8	0	0	1	Virginica

**Table 2:** Estimated normal distribution parameters for evidential Iris data set.

Attributes	Setosa		Versicolor		Virginica	
	μ	σ	μ	σ	μ	σ
SL	5.0031	0.3591	5.9183	0.5419	6.6307	0.6300
SW	3.3822	0.4264	2.7812	0.3201	3.0119	0.2765
PL	1.4640	0.1718	4.3066	0.4961	5.5684	0.5591
PW	0.2408	0.1011	1.3273	0.2074	2.0495	0.2522

**Table 3:** Generated BBAs of selected instance.

Attributes	BBAs
	$m\left(\left\{Setosa\right\}\right)=0.7996$
SL	$m\left(\left\{Setosa,Versicolor\right\}\right)=0.1757$
	$m\left(\left\{Setosa,Versicolor,Virginica\right\}\right)=0.0247$
	$m\left(\left\{Setosa\right\}\right)=0.6904$
SW	$m\left(\left\{Setosa,Virginica\right\}\right)=0.2329$
	$m\left(\left\{Setosa,Versicolor,Virginica\right\}\right)=0.0767$
	$m\left(\left\{Setosa\right\}\right)=1$
PL	$m\left(\left\{Setosa,Versicolor\right\}\right)=0$
	$m\left(\left\{Setosa,Versicolor,Virginica\right\}\right)=0$
	$m\left(\left\{Setosa\right\}\right)=1$
PW	$m\left(\left\{Setosa,Versicolor\right\}\right)=0$
	$m\left(\left\{Setosa,Versicolor,Virginica\right\}\right)=0$

**Table 4:** Classification accuracy on UCI data sets with 90% vacuousness level.

	Iris	Wine	Ionosphere	Balance Scale	Breast Cancer	Sonar
C4.5	0.800	0.6814	0.7221	0.6134	0.8031	0.5247
OBE tree	0.5846	0.4038	0.5775	0.6305	0.7751	0.5553
LBE tree	0.8326	0.6026	0.7763	0.5954	0.8732	0.5793
IBE tree	0.8288	0.6386	0.7797	0.6282	0.8793	0.5841
label voting OBE Random Forest	0.7864	0.4897	0.7404	0.7453	0.8745	0.5611
label voting LBE Random Forest	0.9020	0.8594	0.8555	0.7127	0.9319	0.6149
mass combination LBE Random Forest	0.8989	0.8295	0.8182	0.7138	0.9223	0.6115
label voting IBE Random Forest	0.9053	0.8940	0.8608	**0.7454 **	**0.9338**	0.6120
mass combination IBE Random Forest	**0.9174**	**0.9082**	**0.8647**	0.6891	0.9330	**0.6236**

**Table 5:** Classification accuracy on UCI data sets with 90% imprecision level.

	Iris	Wine	Ionosphere	Balance Scale	Breast Cancer	Sonar
C4.5	0.8000	0.6814	0.7221	0.6134	0.8031	0.5247
OBE tree	0.6233	0.5382	0.6786	0.6746	0.8605	0.5841
LBE tree	0.9093	0.7719	0.7858	0.6146	0.8979	0.6303
IBE tree	0.9040	0.7899	0.7892	0.6381	0.9051	0.6413
label voting OBE random forest	0.8647	0.6208	0.8552	0.7536	0.9257	0.6351
label voting LBE random forest	0.9473	0.9124	0.8621	0.7483	0.9359	0.6630
mass combination LBE random forest	0.9327	0.9118	0.8259	0.7437	0.9262	0.6635
label voting IBE random forest	**0.9467 **	0.9219	0.8684	**0.7709**	0.9364	0.6572
mass combination IBE random forest	0.9447	**0.9326**	**0.8755**	0.7237	**0.9399**	**0.6702**

**Table 6:** Classification accuracy on UCI data sets with 90% uncertainty level.

	Iris	Wine	Ionosphere	Balance Scale	Breast Cancer	Sonar
C4.5	0.8000	0.6814	0.7221	0.6134	0.8031	0.5247
OBE tree	0.8847	0.7792	0.8219	0.7387	0.9121	0.6851
LBE tree	0.9400	0.8180	0.7937	0.6235	0.9120	0.6803
IBE tree	0.9513	0.8399	0.7955	0.6342	0.9278	0.6784
label voting OBE random forest	0.9513	0.9590	**0.9293 **	**0.8233**	0.9381	**0.7577**
label voting LBE random forest	0.9547	0.9489	0.8803	0.7651	0.9344	0.7370
mass combination LBE random forest	0.9560	0.9478	0.8390	0.7800	0.9225	0.7404
label voting IBE random forest	**0.9567**	0.9528	0.8826	0.7829	0.9376	0.7346
mass combination IBE random forest	**0.9567**	**0.9596**	0.8875	0.7496	**0.9394**	0..7433

## Data Availability

Not applicable.
